# *Wolbachia* Affects Reproduction and Population Dynamics of the Coffee Berry Borer (*Hypothenemus hampei*): Implications for Biological Control

**DOI:** 10.3390/insects8010008

**Published:** 2017-01-11

**Authors:** Yobana A. Mariño, José C. Verle Rodrigues, Paul Bayman

**Affiliations:** 1 Department of Biology, University of Puerto Rico—Río Piedras, San Juan, PR 00931, USA; bayman.upr@gmail.com; 2 Center for Excellence in Quarantine and Invasive Species, Agricultural Experimental Station—Río Piedras, Department of Agroenvironmental Sciences, University of Puerto Rico—Mayagüez, 1193 Calle Guayacán, San Juan, PR 00926, USA; jose_carlos@mac.com

**Keywords:** biological control, coffee, coffee berry borer, *Hypothenemus hampei*, population dynamics, sex ratio, *Wolbachia*

## Abstract

*Wolbachia* are widely distributed endosymbiotic bacteria that influence the reproduction and fitness of their hosts. In recent years the manipulation of *Wolbachia* infection has been considered as a potential tool for biological control. The coffee berry borer (CBB), *Hypothenemus hampei*, is the most devastating coffee pest worldwide. *Wolbachia* infection in the CBB has been reported, but until now the role of *Wolbachia* in CBB reproduction and fitness has not been tested. To address this issue we reared the CBB in artificial diets with and without tetracycline (0.1% w/v) for ten generations. Tetracycline reduced significantly the relative proportion of *Wolbachia* in the CBB microbiota from 0.49% to 0.04%. This reduction affected CBB reproduction: females fed with tetracycline had significantly fewer progeny, lower fecundity, and fewer eggs per female. Tetracycline also reduced the population growth rate (λ), net reproductive rate (*R*_0_), and mean generation time (T) in CBB; the reduction in population growth was mostly due to variation in fertility, according to life time response experiments (LTREs) analysis. Our results suggest that *Wolbachia* contribute to the reproductive success of the CBB and their manipulation represents a possible approach to CBB biocontrol mediated by microbiome management.

## 1. Introduction

*Wolbachia* is the most-widely studied genus of endosymbiotic bacteria that infect insects. *Wolbachia* species are cytoplasmically inherited. They are known as reproductive parasites; since they manipulate the reproduction of their hosts in order to enhance their own transmission [1,2,3]. Reproductive manipulations include four mechanisms; cytoplasmic incompatibility, parthenogenesis, feminization, and male killing [4,5,6,7]. However, the relation of *Wolbachia* and their insect hosts also can be mutualistic; several studies have shown that infection with *Wolbachia* may be necessary for the normal reproduction of their hosts [8,9,10,11,12,13,14]. For example, in some species of beetles (Family Curculionidae) the elimination of *Wolbachia* with antibiotics significantly reduced fecundity, and in some cases eggs laid by cured females were not viable [11,13].

The potential role of *Wolbachia* in biological control has received attention in recent years. Infection with *Wolbachia* can be manipulated to increase populations of beneficial insects such as parasitoids or to reduce populations of pests [2,15].

The coffee berry borer (CBB) *Hypothenemus hampei* (Ferrari), (Coleoptera: Curculionidae) is the most devastating pest of coffee worldwide [16,17,18,19]. CBB females tunnel through the fruit until they reach the endosperm, where they oviposit [16,19]. The entire life cycle occurs inside the fruit and the only stage susceptible to CBB management is when the fertilized females leave the fruit to search for another fruit to colonize. This cryptic life cycle and resistance to insecticides such as endosulfan make CBB control extremely difficult and expensive.

Infection of CBB with *Wolbachia* has been reported in adult females from several countries [20]. It is thought to induce cytoplasmic incompatibility and to distort the CBB’s sex ratio towards females [20]. However, the effect of elimination of *Wolbachia* on sex ratio and other reproductive traits has not been tested in CBB. The objectives of this study were to determine the infection status of *Wolbachia* in females from Puerto Rico, and test (by treatment with antibiotics) the effect of removal of *Wolbachia* on CBB reproduction, sex ratio, and population dynamics.

## 2. Materials and Methods

### 2.1. Insect Rearing

Coffee berry borers were reared for ten consecutive generations on the Cenibroca artificial diet [21]. Approximately 10 mL of diet was placed in sterile, clear plastic vials (23 mm × 92 mm, with a plug of bonded dense-weave cellulose acetate, Genessee Scientific, San Diego, CA, USA). Insects were reared in the dark in a growth chamber (Model 818, Thermo Scientific, Dubuque, IA, USA) at 25 °C (±1 °C) and 80%–96% relative humidity. Colonies were maintained in the Center for Excellence in Quarantine and Invasive Species at University of Puerto Rico Agricultural Experimental Station in Río Piedras, PR, USA.

Adult CBB females were collected from infested fruits from arabica coffee in Adjuntas, Puerto Rico (18°10′42.4′′ N, 66°44′36′′ W, 527 m above sea level (masl)). Fruits were carefully dissected with aid of a stereoscope, and females were removed and surface-disinfected by submersion in 1% sodium hypochlorite for 1 min, followed by two rinses with sterile distilled water. For all generations three females were placed in each vial. After each generation adult females were removed from the diet and mixed (randomized) before disinfection and transferal to fresh diet to begin the next generation.

### 2.2. Antibiotics Treatment

For direct and continuous feeding of larvae and adults, tetracycline hydrochloride and penicillin G (Fisher Bioreagents, Pittsburgh, PA, USA) 0.1% (w/v) were added to the Cenibroca diet. Tetracycline is efficient at removing *Wolbachia* [14,22,23]; penicillin is not active against *Wolbachia* [3,24,25] but sometimes is used to control bacterial growth in insect diets; it was used as a parallel control. An additional control group received no antibiotics.

### 2.3. Detection of Wolbachia

DNA was extracted from the entire bodies of adult females obtained from infested fruits in Adjuntas, Puerto Rico, and from laboratory colonies reared on Cenibroca diet with and without antibiotics. Insects were conserved dry and stored in the freezer at −20 °C; total DNA was extracted using the DNeasy Plant Mini Kit (Qiagen Sciences, Hilden, Germany).

The presence of *Wolbachia* in the CBB females was determined by amplification of part of the *wsp* gene (primers wsp-F: 5′ GGGTCCAATAAGTGATGAAGAAAC 3′ and wsp-R: 5′ TTAAAACGCTACTCCAGCTTCTGC 3′ [26] with annealing at 58 °C. Positive controls for PCRs were total DNA of wild *Drosophila melanogaster* (Meigen) (known to carry *Wolbachia*) and negative controls were dH_2_O.

PCR products were cleaned on QIAquick columns (Qiagen, Inc., Hilden, Germany) and sequenced in both directions in the University of Puerto Rico Sequencing and Genotyping Facility (UPR-SGF). The resulting sequences were assembled, manually examined for errors using CodonCode Aligner (version 5.1.5; CodonCode Crop., Centerville, SD, USA), and BLASTed in GenBank to compare the CBB sequences with other *Wolbachia* sequences.

#### 2.3.1. Phylogenetic Classification in *Wolbachia* Supergroups

Phylogenetic analysis was used to assign sequences to *Wolbachia* supergroups. Three *Wolbachia wsp* sequences from this study were compared to GenBank reference sequences from 41 species of insects, including CBB [20]. These 41 sequences were selected from *Wolbachia* supergroups A and B (Table A1) and the sequences from this study were deposited in GenBank (KX436087, KX436088, and KX436089). Sequences were manually aligned with ClustalW in Mesquite [27]. A phylogenetic tree was constructed with Mega 6.0 [28] using neighbor joining, a maximum composite likelihood model, and bootstrap analysis with 1000 replications.

#### 2.3.2. Relative Proportion of *Wolbachia* in the CBB Microbiota

Relative proportions of *Wolbachia* in the microbiota of adult females from control and tetracycline diets (after eleven generations) were determined using Illumina-based 16S typing. Briefly, pooled DNA from five females, with four replicates per treatment, was extracted using DNeasy Plant Mini Kit (Qiagen Sciences). Bacterial 16S rRNA gene was amplified using the hypervariable region V4 with primers 515-F and 806-R [29] with the addition of barcoding sequences and Illumina (Illumina Inc., San Diego, CA, USA) adapters. Sequencing was done on the Illumina MiSeq platform at UPR-SGF. Sequences were assigned to *Wolbachia* using OTU (Operational Taxonomic Units) tables generated with QIIME (Quantitative Insights Into Microbial Ecology) [30]. This experiment is described in detail by Mariño [31].

### 2.4. Effect of Antibiotic Treatments on CBB Reproduction and Sex Ratio

For each generation eggs, larvae, pupae, juveniles, and adults in 20 vials per treatment were counted by carefully dissecting the diet from each tube. The sex ratio was calculated as the number of CBB females divided by the number of males. Sex was determined for adult and juveniles by visual inspection of the body and wing shape and size; males are smaller than females and have reduced degenerative wings [16].

To determine the effect of the elimination or reduction of *Wolbachia* on CBB fecundity, females from the fifth generation of the three treatments were transferred individually to vials with artificial diet (*n* = 50 per treatment); oviposition and number of eggs per female were registered daily for two months.

### 2.5. Effect of Antibiotic Treatment on Life Table Parameters of the CBB

Five cohorts of 20 eggs each were established for each type of diet. Eggs were maintained in the dark at 25 °C (±1 °C) and a relative humidity of 80%–96%. Each cohort was evaluated daily for 50 days at 24-h intervals to determine CBB survival and the transitions to the next stages of development. Also, the development time from egg to adult was determined.

For population dynamics analysis, the basic life cycle for the CBB was defined as egg, larva, pre-pupa, pupa, juvenile, and adult. The probabilities of the projection matrix were calculated at intervals of six days: (*Gi*) probability of growth or molt between stages, (*Pi*) probability of surviving and remaining in the same stage, and (*Fi*) fertility of adult females; these values were based on the data obtained in this study for oviposition of fifty females. The probability of adult survival was estimated as 0.99, because observed mortality of adults was very rare once they emerged; several studies also reported low natural mortality rates in field and laboratory experiments [32,33,34].

Based on the projection matrix (Equation (1)) for each cohort in the three types of diet, we calculated: the population growth rate (λ), the net reproductive value (*R*_0_), and mean generation time (T) [35].

A life table response experiment (LTRE) analysis was done to determine the contribution of each life cycle transition in the observed reduction of the population growth rate (λ) in CBB populations treated with tetracycline (0.1% w/v) compared with the other two diets (control and penicillin).

### 2.6. Data Analysis

The abundance of individuals in various stages of development of the coffee berry borer and the number of eggs laid per female were estimated using a log-linear model (GLM) with a Poisson distribution; type of diet was defined as an independent variable. Chi-square analysis was used to compare differences in the numbers of females that oviposited among types of diet and to determine differences in the number of reads of *Wolbachia* found in adult females between tetracycline vs. control diets. One way ANOVAs followed by post-hoc Tukey tests (significance level *p* = 0.05) were used to test differences among types of diet on sex ratio, life table population parameters, developmental time from egg to adult, and the duration of each stage of development. The package popbio (version 2.4.3) was used to calculate all life table parameters and perform the LTRE analysis [36]. All analyses were performed in R [37].

## 3. Results

### 3.1. Detection of Wolbachia

A ≈ 750-bp fragment was amplified and sequenced from the *Wolbachia wsp* gene from CBB field-collected and females reared in the laboratory on the Cenibroca diet. The positive control (*Drosophila*) amplified a band of similar size; no product was amplified in any of the negative controls. Sequences of seven *wsp* fragments exhibited 100% sequence similarity to *Wolbachia* from *Drosophila melanogaster* (FJ403332).

**Figure 1:**
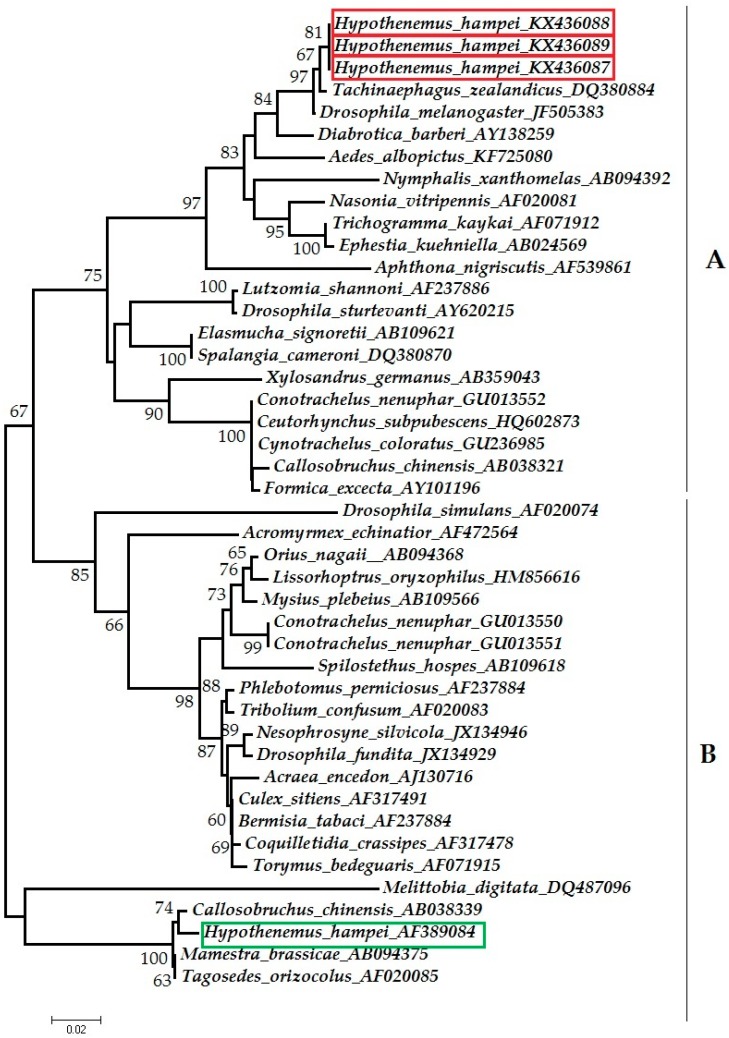
Neighbor-joining tree showing placement of *wsp* sequences of *Wolbachia* from *Hypothenemus hampei* in *Wolbachia* supergroups A and B. Sequences from this study are shown in red boxes; the sequence from *H. hampei* from Vega et al. [20] is shown in a green box. Reference sequences from endosymbionts of 41 species of insects are included. The tree was constructed using the Maximum Composite Likelihood model and midpoint rooted. Bootstrap probabilities >50% are shown at the nodes. GenBank accession numbers are shown.

The *wsp* sequences from *H. hampei* from Puerto Rico grouped with those of *Wolbachia* supergroup A. The sequences from CBB formed a separate subclade within supergroup A, closely related to one from a symbiont of the parasitoid *Tachinaephagus zealandicus* (Ashmead) (DQ380884) [38] (Figure 1). In contrast, a *wsp* consensus sequence from *H. hampei* from India and Brazil (AF389084) grouped with supergroup B [20], and with others from the adzuki bean beetle *Callasobruchus chinensis* (Linnaeus) (AB038339) [26].

### 3.2. Effect of Antibiotic Treatment on *Wolbachia* Infection and CBB Reproduction

After eleven generations on diet with tetracycline *Wolbachia* was still detected in CBB. However, the relative proportion of *Wolbachia* in the CBB microbiota was significantly lower in females from the diet with tetracycline than from the control diet (0.04% vs. 0.49%, χ ^2^ = 6265.9, df = 1, *p* < 0.001).

**Figure 2:**
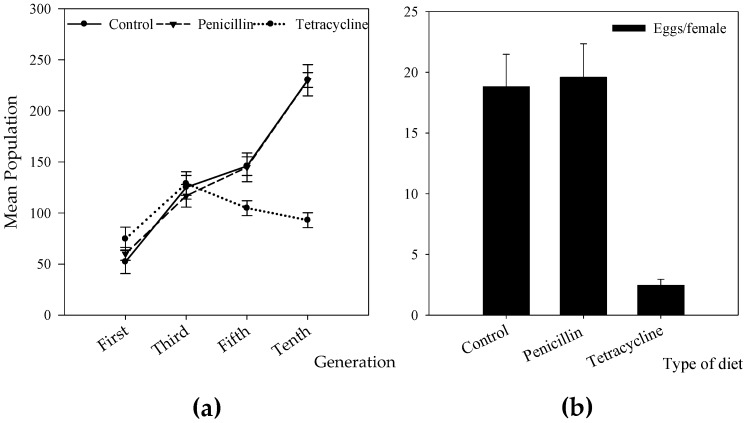
Effects of antibiotics on *Hypothenemus hampei* population growth and fecundity. (**a**) Total number of individuals (including eggs, larvae, pupae, juveniles, and adults) for F_1_, F_3_, F_5_, and F_10_ generations (**b**) Fecundity (number of eggs per female). Means ± SE are shown.

The log-linear model gave a good fit for the effects of diet on CBB total population (χ_7_^2^ = 225, *p* < 0.0001). In general, the estimated population was significantly higher in control and penicillin diets than in tetracycline (Table A2). The estimated population per generation increased independently of type of diet (Table A2). After the F_3_ generation females reared with tetracycline produced significantly fewer progeny, and in the F_10_ generation less than half as many individuals were produced compared with control and penicillin diets (Figure 2A).The estimated abundance of individuals at all stages of development varied significantly among diets (GLM, Poisson errors, eggs *Z* = 232.2, *p* < 0.0001; larvae *Z* = 194.7, *p* < 0.0001, pupae *Z* = 107.9, juveniles *Z* = 52.20, *p* < 0.0001, and adults *Z* = 295.4, *p* < 0.0001). In general, the number of individuals in all stages of development was significantly lower in diets with tetracycline than in controls.

The proportion of females that oviposited was significantly lower in diet with tetracycline (38% in tetracycline, 68% in control, and 66% in penicillin, χ^2^ = 11.50, df = 2, *p* = 0.003). Also, the number of eggs per female was significantly lower in diets with tetracycline: on average, each female laid 3.45 eggs compared with 18.83 in control and 19.58 in penicillin (GLM, Poisson errors, eggs *Z* = 86.79, *p* < 0.0001) (Figure 2B).

Significant differences were found in sex ratio between diets (*F* = 6.48, df = 2, *p* = 0.002). The sex ratio was less skewed towards females in tetracycline diet than in control and penicillin diets (19.4:1 for tetracycline, 24.1:1 for control, and 26.5:1 for penicillin). These differences in sex ratio were significant from the F3 generation and all subsequent generations; no differences were observed in F1 and F2 (Table 1). However, no differences were observed in the number of males among diets (control vs. penicillin: *Z* = 1.32, *p* = 0.25; control vs. tetracycline: *Z* = −0.52, *p* = 0.59).

### 3.3. Effect of Antibiotic Treatment on Life Table Parameters of the CBB

The duration of the juvenile stage was significantly longer in tetracycline than in control and penicillin. However, the duration of the remaining stages did not differ between diets; also, the egg-to-adult developmental time did not differ significantly between diets, but was slightly higher for individuals reared with tetracycline (Table 2).

Life table parameters for the three diets are presented in Table 3. Net reproductive value *R*_0_ differed significantly among diets (Table 3). The net reproductive value (*R*_0_) was 23 females/female for tetracycline, 51 for control, and 45 for penicillin. In other words, a female on these diets produced on average 23, 51, and 45 new females respectively in each generation.

**Figure 3:**
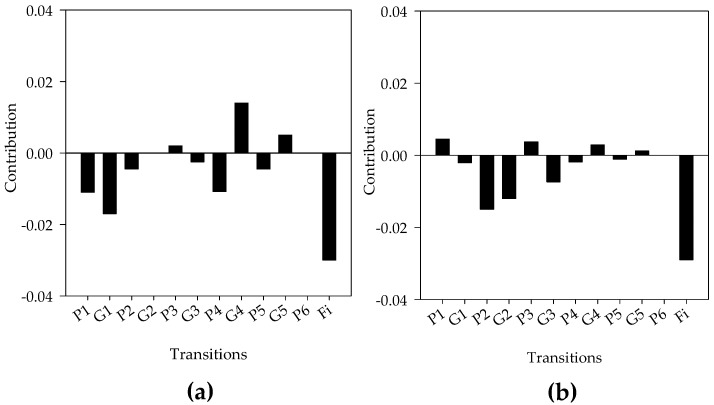
Life table response experiment (LTRE) analysis on the coffee berry borer *Hypothenemus hampei* treated with tetracycline (0.1% w/v) compared with control (without antibiotics) and penicillin (0.1% w/v) treatments. The bars show the contribution of each life cycle transition to the reduction in population growth rate (λ) when comparing diets. (**a**) Control vs. Tetracycline and (**b**) Penicillin vs. Tetracycline. *Gi*: probability of transition between stages, *Pi*: probability of surviving and remaining in the same stage, *Fi*: fertility of adult females. (1) Eggs, (2) Larvae, (3) Pre-pupae, (4) Pupae, (5) Juveniles, (6) Adults.

For all diets the population growth rate was >1, but populations treated with tetracycline had a slightly lower λ; the LTRE showed that reduction in fecundity of adult females feeding with tetracycline made the largest contribution to the reduction in λ (Figure 3). Although differences in the mean generation time (T) were not significant, tetracycline treatment increased the time by 6.2 days, which means that females reared with tetracycline will produce fewer generations per year (Table 3).

### 3.4. Formatting of Mathematical Components

For the projection matrix, variations in the size and age structure of a population **N** through time *t* and *t*+1 were computed from the equation:

(1) $$N_{t+1}={\mathrm{AN}}_{t}$$

where **A** is a population matrix and **N** is a vector describing the age of structured population. Thus, the projection matrix was:

(2) $${\left[\begin{array}{c} n_{1}\\ n_{2}\\ n_{3}\\ n_{4}\\ n_{5}\\ n_{6} \end{array}\right]}_{t+1}A=\left[\begin{array}{cccccc} P_{1} & 0 & 0 & 0 & 0 & F_{1}\\ G_{1} & P_{2} & 0 & 0 & 0 & 0\\ 0 & G_{2} & P_{3} & 0 & 0 & 0\\ 0 & 0 & G_{3} & P_{4} & 0 & 0\\ 0 & 0 & 0 & G_{4} & P_{5} & 0\\ 0 & 0 & 0 & 0 & G_{5} & P_{6} \end{array}\right]\times N={\left[\begin{array}{c} n_{1}\\ n_{2}\\ n_{3}\\ n_{4}\\ n_{5}\\ n_{6} \end{array}\right]}_{t}$$

## 4. Discussion

### 4.1. Wolbachia in Insects and Status in the CBB

*Wolbachia* are widespread and common insect endosymbionts and have been detected in more than 66% of insect species tested [7,39]. In many species, infection rate is variable; infected individuals within a species ranges from <3% to 100% [40,41]. Also, the titer of *Wolbachia* can be as low as <0.01 or as high as >1200 cells per insect cell [42,43] (though *Wolbachia* are not uniformly distributed throughout the insect but concentrated in special organs). Furthermore, there are populations with high infection frequency of *Wolbachia*, but titer per individual is low. This was demonstrated in the bark beetle *Pytogenes chalcographus* (Linneaus), which had infection frequencies of 85%–100%, but the titer per cell was <0.5 [44]. It is not clear if there is a threshold titer for *Wolbachia* to affect insects.

In the case of the CBB, there are no published studies that reported the infection rate or titer. Vega et al. [20] detected *Wolbachia* in ten of fifteen samples, which could suggest a high infection frequency, but they did not report how many individuals were tested per sample. The data reported here are averages from pooled samples, so there is no way of detecting variation in titer among individuals.

In this study the success rate for amplifying *Wolbachia* using conventional PCR was <10%. 16S sequencing showed a low relative proportion of *Wolbachia* in the CBB microbiota of adult females reared in artificial diets and collected from the field, 0.49% and 0.16% respectively [31]. A low titer of *Wolbachia* in the CBB may explain the difficulty of amplification, as Vega et al. [20] suggested. Yet the data presented here suggest that even a low titer of *Wolbachia* can have a significant effect on insect reproductive biology. Furthermore, the difference between treatments with 0.49% relative proportion of *Wolbachia* in females from control diet and 0.04% in females from tetracycline suggests the possibility of a dosage-dependent effect.

### 4.2. Placement of CBB Symbionts in Wolbachia Supergroups

For *Wolbachia* classification the *wsp* gene was preferred by Zhuo et al. [5]; *wsp* is evolving more rapidly than 16S rDNA and *ftsZ* genes. According to phylogenetic studies using these three genes, the genus *Wolbachia* is divided into eight supergroups (A–H) [45,46,47,48]. The supergroups A and B are exclusively found in arthropods and are the most common; C and D are found in nematodes. The remaining supergroups (E–H) have been proposed more recently and may be less common [46,47,49]. However, the diversity and the number of supergroups in *Wolbachia* are increasing as different genes are explored: for example, eleven supergroups were recognized when the additional genes *gltA* and *groEL* were included [50].

The term supergroup is used to define a clade of phylogenetically related strains [5,49]; the *Wolbachia* in CBB females from Puerto Rico belonged to supergroup A, while the ‘consensus sequence’ from India and Brazil belonged to supergroup B [20] (Figure 1). The subdivision of *Wolbachia* into supergroups was done in response to its high genetic diversity compared with other endosymbionts [49]. However, to our knowledge there are no studies demonstrating functional differences between *Wolbachia* supergroups A and B. More studies should be conducted to determine the diversity and distribution of *Wolbachia* strains in the CBB, and to elucidate the significance of this diversity in CBB biology.

### 4.3. Antibiotic Treatments and Their Effect on CBB Reproduction and Fitness

The effect of tetracycline on insect reproduction and fitness of *Wolbachia*-infected insects can result from curing or reducing *Wolbachia* infection, but could also reflect direct toxicity on host physiology [14]. It is also possible that tetracycline caused other changes in the microbiota unrelated to *Wolbachia*. These possible explanations are explored further in Mariño [31]. To reduce the possibility that the observed effects on CBB were a result of direct antibiotic toxicity or other changes in the microbiota, we included a parallel treatment with penicillin G; penicillin G is not effective against *Wolbachia* [3,24,25]. Tetracycline and penicillin differ in their modes of action and ranges of activity; however, both antibiotics can affect directly the fecundity and survival of insects [51,52,53]. Moreover, in this study penicillin did not affect reproduction in the CBB, the reverse of what would occur if the observed effects were due to toxicity. It also reduces (but no eliminates) the possibility that the results reflect the effect of antibiotics on nontarget groups of bacteria, because penicillin has a narrower spectrum of activity than tetracycline.

Our results showed that females reared on penicillin and control diets produced a similar number of individuals in all stages of development, and there were no significant effects of penicillin on reproduction and life table parameters (Figure 2 and Table 1, Table 2 and Table 3). *Pseudomonas fulva* (Iizuka and Komagona) is involved in caffeine degradation in the CBB [54]. When *P. fulva* was eliminated from adult females fed with a mix of three antibiotics including tetracycline, there was a significant reduction in the number of eggs in females fed with the antibiotics [54]. However, reinoculation with *P. fulva* did not restore the normal reproduction of females. This suggests that *P. fulva* was not responsible for the reduced egg production, and *Wolbachia* may have been responsible. This reduced reproduction with tetracycline may be due to the effect of reduction of *Wolbachia* (though this possibility was not mentioned by Ceja-Navarro et al. [54]; *Rickettsia*, which includes other endosymbiotic bacteria that affect insect reproduction, was recently reported in the CBB [54], but, we did not detect it in CBB from Puerto Rico [31].

Treatment with tetracycline is a well-known method to eliminate *Wolbachia* from hosts [11,14,55] and test its effect on the reproduction of its hosts. However, our treatment with tetracycline 0.1% (w/v) did not eradicate it, even after eleven generations of continuous exposure. Tetracycline 0.1% or (even less) was enough to remove *Wolbachia* from other hosts [22,55,56,57]. Even though the infection was not completely cured, our results strongly suggest a relationship between *Wolbachia* and the successful reproduction of CBB females. After three generations, the reproduction of females treated with tetracycline started to decrease significantly; by the tenth generation their progeny was less than half compared to females reared in penicillin and control diets (Figure 2A). Also, females treated with tetracycline produced significantly fewer eggs (Figure 2B).

This fertility reduction agrees with data from other beetles in the family Curculionidae. In the date stone beetle (*Coccotrypes dactyliperda* Fabricius) mated females fed with 3% tetracycline produced significantly fewer eggs than control females [11]. Similarly, in the rice water weevil (*Lissorhoptrus oryzophilus* Kuschel) females treated with 0.25%–2.5% tetracycline produced fewer eggs and none of these eggs were viable [13]. The authors of both studies attributed the reduction in fecundity in these species to the elimination of endosymbiotic bacteria, mainly *Wolbachia*.

*Wolbachia* are commonly known as reproductive parasites, due their capacity to manipulate the reproduction of their hosts in different ways to enhance their own transmission [1]. However, some *Wolbachia* can be mutualists [6], involved in nutritional mechanisms reported in *Drosophila melanogaster* [58] and recently in *Cimex lectularius* (Linneaus) [59] and *Aedes albopictus* (Skuse) [60]. Our results suggest that *Wolbachia* plays an important role in CBB reproduction.

### 4.4. Sex Ratio

Infection by endosymbiotic bacteria has been shown to be associated with female-biased sex ratios [1,22,61,62,63]. In some insects, females cured with antibiotics produce more males or only males [64,65,66,67,68,69].

In the only previous study of *Wolbachia* in CBB, Vega et al. [20] suggested that *Wolbachia* plays a role in the female-biased sex ratio observed in the CBB. Our results showed that *Wolbachia*’s effects on the sex ratio are at best indirect: females treated with tetracycline produced significantly fewer daughters than females from controls, but tetracycline did not affect significantly the number of males produced (Table 1). The less skewed sex ratio observed with tetracycline was more a consequence of the reduction in the number of females than the production of more males.

Contrary to Vega et al. [20], we suggest that *Wolbachia* is not involved in the sex determination of CBB; a significant increase in the number of males in progenies from females treated with tetracycline would be the expected result if *Wolbachia* were involved in sex determination. Other mechanisms, mainly the sex determination systems haplodiploidy and functional haplodiploidy, are known to induce highly female-biased sex ratios [22,70]. Functional haplodiploidy is the sex determination system described for the CBB [71]; in this system, the sex ratio distortion is a consequence of high male mortality, which occurs due to the loss of paternal chromosomes [9,12].

However, this result needs to be confirmed through the production of *Wolbachia*-cured lines. The best method to assess the impact of *Wolbachia* on sex determination in its hosts is to compare an infected line with a *Wolbachia*-cured line [72]. It is also important to determine the minimum dose of tetracycline needed to completely cure *Wolbachia* infections; 3% (w/v) tetracycline eliminated endosymbiotic bacteria, including *Wolbachia*, from *Coccotrypes dactyliperda* (Coleoptera: Curculionidae) [11]. However, such high concentrations also exacerbate toxicity issues. Alternatively, another antibiotic like rifampicin could be used; rifampicin is also effective against *Wolbachia* [14,24].

Relatively few studies have measured the effect of *Wolbachia* in arthropod population dynamics. Demographic studies allow comparing the growth potential for a population under different conditions; life time response experiments (LTREs) deconstruct differences on population growth into contributions from each life cycle transition [73]. In the case of insect pests like the CBB, this type of analysis could provide insight into which specific life cycle transition could be targeted for management.

*Wolbachia*-free populations of *Liposcelis tricolor* (Badonnel), (Psocoptera: Liposcelididae) had lower performance in all life parameters evaluated: intrinsic rate of increase *rm*, net reproductive value *R*_0_, and mean generation time. Similarly, our data showed that tetracycline reduced the values of all life table parameters evaluated in the CBB. The LTRE analysis identified the fecundity of adult females as the stage contributing most to the reduction of λ (Figure 3). Taken together, these data suggest that *Wolbachia* are important for successful reproduction of the CBB.

## 5. Conclusions

In this study we detected *Wolbachia* supergroup A in adult CBB females from Puerto Rico. The relative proportion of *Wolbachia* in the CBB microbiome is very low, less than 1%.

*Wolbachia* appears to contribute to reproductive success in the CBB: antibiotic treatment significantly reduced the proportion of *Wolbachia*, and had negative effects on reproduction and population dynamic of CBB. This result opens the door to the possibility of CBB biocontrol mediated by manipulating *Wolbachia* infection, either by reducing *Wolbachia* infection or encouraging growth of competing microorganisms in the CBB microbiota. The effects and mechanisms of *Wolbachia* on CBB appear to be complex, but our results show they have pronounced effects on the reproductive biology of this important pest. Considering the CBB’s importance, the possibility of biological control approaches that exploit this new information deserves further investigation.

## Figures and Tables

**Table 1:** Number of females, males, and sex ratio of *Hypothenemus hampei* reared in artificial diets with and without antibiotics; Means ± SE and F: M ratio per vial for F_1_, F_5_, and F_10_ generations.

**Diet**	**Generation (F_1_)**		
	**Females**	**Males**	**Sex Ratio**
control	20.8 ± 1.8	2.4 ± 0.3	10.2 ± 0.8 a
penicillin	20.6 ± 3.4	2.8 ± 0.4	8.80 ± 1.2 a
tetracycline	17.5 ± 2.5	3.5 ± 0.4	10.3 ± 1.7 a
Statistics	*Z* = 61.0/*p* = 0.04 ^a^ *	*Z* = 7.29/*p* < 0.77 ^a^	*F* = 0.4/*p* = 0.66 ^b^
	**Generation (F_5_)**		
	**Females**	**Males**	**Sex Ratio**
control	62.0 ± 4.9	9.4 ± 0.6	31.0 ± 2.4 b
penicillin	39.1 ± 4.8	5.5 ± 0.7	19.5 ± 2.4 a
tetracycline	27.1 ± 3.5	6.2 ± 0.7	13.5 ± 1.7 a
Statistics	*Z* = 1.05/*p* < 0.001 ^a^ ***	*Z* = 30.5/*p* < 0.001 ^a^ ***	*F* = 16.13/*p* < 0.001 ^b^ ***
	**Generation (F_10_)**		
	**Females**	**Males**	**Sex Ratio**
control	84.2 ± 5.1	11.3 ± 0.8	42.1 ± 2.5 b
penicillin	98.3 ± 8.6	10.9 ± 0.8	49.2 ± 4.3 b
tetracycline	52.5 ± 7.2	9.1 ± 1.1	26.5 ± 3.6 a
Statistics	*Z* = 182.0/*p* < 0.001 ^a^ ***	*Z* = 36.45/*p* = 0.74 ^a^	*F* = 10.81/*p* = 0.0001 ^b^ ***

^a^ = Values based on generalized linear models; ^b^ = Values based on ANOVA tests. Means followed by the same letter within a column are not significantly different (*p* = 0.05, Post-hoc Tukey’s test). *** *p* < 0.001; ** *p* < 0.01; * *p* < 0.05; *p* < 0.1.

**Table 2:** Developmental time in days (Mean ± SE) for life stages of the coffee berry borer *Hypothenemus hampei* reared in artificial diet with and without antibiotics.

Diet	Development Time (Days)					
	Egg	Larva	Pre-Pupa	Pupa	Juvenile	Egg to Adult
control	6.08 ± 0.22 **a**	15.22 ± 0.75 **a**	1.94 ± 0.11 **a**	6.37 ± 0.15 **a**	4.05 ± 0.12 **a**	34.41 ± 0.75 **a**
penicillin	6.07 ± 0.31 **a**	15.45 ± 0.59 **a**	1.87 ± 0.07 **a**	6.78 ± 0.18 **a**	4.09 ± 0.19 **a**	34.26 ± 0.61 **a**
tetracycline	6.10 ± 0.21 **a**	15.52 ± 0.74 **a**	2.03 ± 0.09 **a**	6.82 ± 0.26 **a**	4.67 ± 0.24 **b**	35.13 ± 0.94 **a**
Statistics	*F* = 1.91/*p* = 0.15	*F* = 0.89/*p* = 0.41	*F* = 0.86/*p* = 0.42	*F* = 1.59/*p* = 0.20	*F* = 3.34/*p* = 0.01 *	*F* = 0.95/*p* = 0.38

Means followed by the same letter within a column are not significantly different (*p* = 0.05, Post-hoc Tukey’s test). *** *p* < 0.001; ** *p* < 0.01; * *p* < 0.05; *p* < 0.1.

**Table 3:** Life table parameters of the coffee berry borer *Hypothenemus hampei* reared in artificial diet with and without antibiotics.

Diet	Life Table Parameters		
	λ	*R*_0_	T
control	1.11 ± 0.01 **a**	51.51 ± 5.72 **b**	39.01 ± 3.13 **a**
penicillin	1.10 ± 0.01 **a**	45.20 ± 8.88 **ab**	39.19 ± 5.39 **a**
tetracycline	1.07 ± 0.01 **a**	23.40 ± 3.43 **a**	45.19 ± 3.05 **a**
Statistics	*F* = 3.13/*p* = 0.08	*F* = 5.29/*p* = 0.02 *	*F* = 0.77/*p* = 0.48

λ = population growth rate; *R*_0_ = net reproductive value; T = mean generation time. Statistical significance based on ANOVA tests. Means followed by the same letter within a column are not significantly different. (*p* = 0.05, Post-hoc Tukey’s test). *** *p* < 0.001; ** *p* < 0.01; * *p* < 0.05; *p* < 0.1.

## Appendix A

**Table A1:** Reference sequences of *Wolbachia* used to determine CBB *Wolbachia* supergroup classification.

Host Insect Species	Order	*Wolbachia* Supergroup	GenBank Accession No.
*Aphthona nigriscutis*	Coleoptera	A	AF539861
*Callosobruchus chinensis*	Coleoptera	A	AB038321
*Ceutorhynchus subpubescens*	Coleoptera	A	HQ602873
*Conotrachelus nenuphar*	Coleoptera	A	GU013552
*Cynotrachelus coloratus*	Coleoptera	A	GU236985
*Diabrotica barberi*	Coleoptera	A	AY138259
*Hypothenemus hampei*	Coleoptera	A	KX436087
*Hypothenemus hampei*	Coleoptera	A	KX436088
*Hypothenemus hampei*	Coleoptera	A	KX436089
*Xylosandrus germanus*	Coleoptera	A	AB359043
*Aedes albopictus*	Diptera	A	KF725080
*Drosophila melanogaster*	Diptera	A	JF505383
*Drosophila sturtevanti*	Diptera	A	AY620215
*Lutzomia shannoni*	Diptera	A	AF237886
*Elasmucha signoretii*	Heteroptera	A	AB109621
*Formica excecta*	Hymenoptera	A	AY101196
*Nasonia vitripennis*	Hymenoptera	A	AF020081
*Spalangia cameroni*	Hymenoptera	A	DQ380870
*Tachinaephagus zealandicus*	Hymenoptera	A	DQ380884
*Trichogramma kaykai*	Hymenoptera	A	AF071912
*Ephesia kuehniella*	Lepidoptera	A	AB024569
*Nymphalis xanthomelas*	Lepidoptera	A	AB094392
*Callosobruchus chinensis*	Coleoptera	B	AB038339
*Conotrachelus nenuphar*	Coleoptera	B	GU013550
*Conotrachelus nenuphar*	Coleoptera	B	GU013551
*Hypothenemus hampei*	Coleoptera	B	AF389084
*Lissorhotrus oryzophilus*	Coleoptera	B	HM856616
*Tribolium confusum*	Coleoptera	B	AF020083
*Culex sitiens*	Diptera	B	AF317491
*Coquilletidia crassipes*	Diptera	B	AF317478
*Drosophila fundita*	Diptera	B	JX134929
*Drosophila simulans*	Diptera	B	AF020074
*Phlebotomus perniciosus*	Diptera	B	AF237884
*Bermisia tabaci*	Hemiptera	B	AF237884
*Nesophrosyne silvicola*	Hemiptera	B	JX134946
*Orius nagaii*	Hemiptera	B	AB094368
*Tagosedes orizocolus*	Hemiptera	B	AF020085
*Mysius plebeius*	Heteroptera	B	AB109566
*Spilostethus hospes*	Heteroptera	B	AB109618
*Acromyrmex echinatior*	Hymenoptera	B	AF472564
*Melittobia digitata*	Hymenoptera	B	DQ487096
*Torymus bedeguaris*	Hymenoptera	B	AF071915
*Acraea encedon*	Lepidoptera	B	AJ130716
*Mamestra brassicae*	Lepidoptera	B	AB094375

**Table A2:** Results of generalized linear model evaluating the relationship between CBB total population and type of diet (control, penicillin 0.1%, and tetracycline 0.1%) and between generations for CBB on diet with tetracycline. *** *p* < 0.001; ** *p* < 0.01; * *p* < 0.05; *p* < 0.1.

Effects	Estimate	Standard Error	Z-Value	*p*
Intercept	4.108	0.0177	231.6	<0.0001 ***
Type of diet (control vs. penicillin)	0.0871	0.0109	7.93	0.0670
Type of diet (control vs. tetracycline)	−0.0804	0.0114	−7.02	<0.0001 ***
Generation (F_1_ vs. F_2_)	0.7764	0.0199	38.91	<0.0001 ***
Generation (F_1_ vs. F_3_)	0.8587	0.0197	43.58	<0.0001 ***
Generation (F_1_ vs. F_4_)	0.8285	0.0197	41.86	<0.0001 ***
Generation (F_1_ vs. F_5_)	0.6950	0.0202	34.38	<0.0001 ***
Generation (F_1_ vs. F_10_)	1.172	0.0188	62.07	<0.0001 ***
